# Influence of PepF peptidase and sporulation on microcin J25 production in *Bacillus subtilis*

**DOI:** 10.1128/spectrum.03748-23

**Published:** 2024-05-23

**Authors:** Guangwen Zhang, Saixiang Feng, Miaomiao Qin, Juan Sun, Yutong Liu, Changqi Luo, Min Lin, Siqi Xu, Ming Liao, Huiying Fan, Zhaoping Liang

**Affiliations:** 1 College of Veterinary Medicine, South China Agricultural University, Guangzhou, China; 2 Key Laboratory of Zoonosis Prevention and Control of Guangdong Province, Guangzhou, China; 3 Guangdong Laboratory for Lingnan Modern Agriculture, South China Agricultural University, Guangzhou, China; 4 Key Laboratory of Veterinary Vaccine Innovation of the Ministry of Agriculture, Guangzhou, China; 5 National and Regional Joint Engineering Laboratory for Medicament of Zoonosis Prevention and Control, Guangzhou, China; 6 Institute of Animal Health, Guangdong Academy of Agricultural Sciences, Guangzhou, China; University of Saskatchewan, Saskatoon, Canada

**Keywords:** microcin J25, peptidase, PepF, *Bacillus subtilis*, sporulation

## Abstract

The lasso peptide microcin J25 (MccJ25) possesses strong antibacterial properties and is considered a potential effective component of bacterial disease treatment drugs and safe food preservatives. Although MccJ25 can be heterologously expressed in *Bacillus subtilis* as we have previously reported, its regulation and accumulation are yet to be understood. Here, we investigated the expression level and stability of MccJ25 in *B. subtilis* strains with disruption in peptidase genes *pepA*, *pepF,* and *pepT*. Oligoendopeptidase F (PepF) was found to be involved in reduction of the production of MccJ25 by degradation of its precursor peptide. In the *pepF* mutant, the MccJ25 reached a concentration of 1.68 µM after a cultivation time exceeding 60 hours, while the wild-type strain exhibited a concentration of only 0.14 µM. Moreover, the production of MccJ25 in *B. subtilis* downregulated the genes associated with sporulation, and this may contribute to its accumulation. Finally, this study provides a strategy to improve the stability and production of MccJ25 in *B. subtilis*.

**IMPORTANCE:**

MccJ25 displays significant antibacterial activity, a well-defined mode of action, exceptional safety, and remarkable stability. Hence, it presents itself as a compelling candidate for an optimal antibacterial or anti-endotoxin medication. The successful establishment of exogenous production of MccJ25 in *Bacillus subtilis* provides a strategy for reducing its production cost and diversifying its utilization. In this study, we have provided evidence indicating that both peptidase PepF and sporulation are significant factors that limit the expression of MccJ25 in B. subtilis. The Δ*pepF* and Δ*sigF* mutants of *B. subtilis* express MccJ25 with higher production yield and enhanced stability. To sum up, this study developed several better engineered strains of *B. subtilis*, which greatly reduced the consumption of MccJ25 during the nutrient depletion stage of the host strain, improved its production, and elucidated factors that may be involved in reducing MccJ25 accumulation in *B. subtilis*.

## INTRODUCTION

Microcins are antimicrobial peptides synthesized by ribosomes and are produced by some Enterobacteriaceae species under the condition of nutrient depletion to kill or inhibit neighboring competitors (1, 2). Microcins are divided into two categories, namely, class I and class II, according to their molecular masses. Class I microcins have mass less than 5 kDa, while that of class II microcins is between 5 and 10 kDa (1). Microcin J25 (MccJ25) is a class I microcin with a unique lasso-like structure (3–5) that inhibits or kills target bacteria (primarily Gram-negative Enterobacteriaceae) by inhibiting bacterial RNA polymerase activity (6) and disrupting the membrane respiratory chain (7). MccJ25 is considered a good alternative to antibiotics because it is neither toxic nor hemolytic at the highest concentration required to inhibit bacterial growth (8–11). The gene cluster encoding MccJ25 is *mcjABCD*, in which the *mcjA* gene encodes for the precursor protein, the *mcjB* and *mcjC* genes encode posttranslational modification enzymes, and the *mcjD* gene encodes the self-immunity factor and secretion protein (12, 13). In the MccJ25-encoding gene cluster of *Escherichia coli*, there are two promoters between the *mcjA* and *mcjB* genes, P*_mcjA_* and P*_mcjBCD_*, which direct the transcription of the *mcjA* and *mcjBCD* genes in the opposite direction, respectively (1, 14, 15). Although there are many literature reports on the expression and production of MccJ25 using *E. coli* (3, 7, 12, 16, 17), this production method, however, poses the issue of endotoxin contamination.

**Fig 1:**
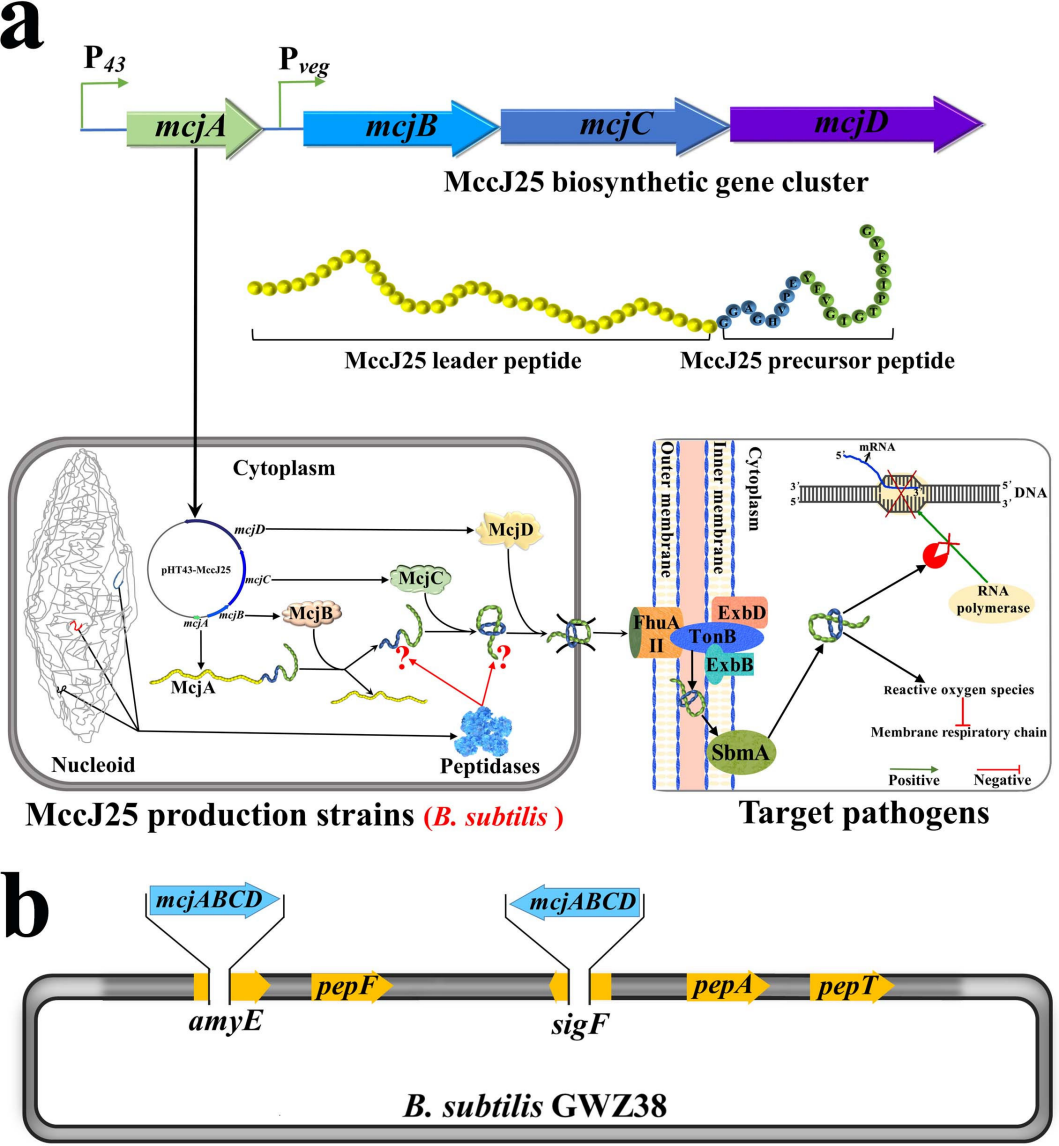
Heterologous production of MccJ25 in *B. subtilis*. (a) Schematic diagram of the biosynthesis of MccJ25 in *B. subtilis* and its bactericidal mechanism. (b) Schematic representation of the genetic modification of the MccJ25-producing *B. subtilis* GWZ38 strain.

*Bacillus subtilis* is considered an ideal host for heterologous protein production due to its well-characterized genetics and metabolic pathways, "generally recognized as safe" (GRAS) status, high-level protein secretion ability, and its remarkable adaptability to extreme conditions. It has been extensively applied in fields such as food biotechnology, medicine, and agriculture (18–20). Recently, we constructed a gene cluster encoding MccJ25 in *B. subtilis,* P*_43_-mcjA*–P*_veg_-mcjBCD* (Fig. 1a), thus establishing a probiotic expression system for MccJ25 (21). In addition, we showed that there are many factors that can limit the production of MccJ25 in *B. subtilis*, including the transcription level of each coding gene, the copy number of the coding gene cluster, and the degradation activity of extracellular proteases (21). In fact, although we have done our best to study and overcome the negative effects of the aforementioned problems, some inexplicable phenomena remain poorly understood. Despite the removal of extracellular proteases in the *B. subtilis* WB800N strain, the production of MccJ25 still rapidly decreases after 36 hours of expression (21). This led to our interest in exploring other factors affecting biosynthesis of MccJ25 in *B. subtilis*.

Peptidases are important tools in the growth and metabolism of various organisms, including *B. subtilis*, and they are classified as exopeptidases and endopeptidases depending on the position of peptide bond cleavage (22). Different classes of peptidases are associated with important biological pathways. Based on similarity, PepA (*yuiE*) is predicted to be an aminopeptidase in *B. subtilis*, but its exact function has not been studied. In *E. coli*, PepA is a well-defined leucyl aminopeptidase with cysteinyl–glycinase activity, which involves glutathione turnover (23). Since glutathione is absent in *B. subtilis*, the pathway of action of this enzyme in *B. subtilis* has not been clearly defined (24). According to gene sequencing analysis, PepF in *B. subtilis* is anticipated to be a cytoplasmic endopeptidase (25). Previous investigations have revealed that overexpression of PepF can hinder the initiation of sporulation in *B. subtilis*, but its specific function in *B. subtilis* physiology remains unclear (26). *B. subtilis* PepT is an aminopeptidase that cleaves the N-terminal amino acid of tripeptides and is homologous to tripeptidases from a variety of different bacterial sources (27). Although studies have demonstrated the high resistance of MccJ25 to proteases (including peptidases) (1), there is no report on the impact of MccJ25 expression on the peptidases PepA, PepF, and PepT in *B. subtilis*.

Spore formation is a strategy used by *B. subtilis* cells to temporarily escape nutrient-poor conditions through dormancy; the cells continuously monitor the nutritional status of the surrounding environment and rapidly respond to the surrounding nutrient environment by germinating and resuming growth (28). Sigma factor F (*sigF*) is a gene required to control the early stages of spore development in *B. subtilis*, and disruption of *sigF* prevents the continuation of the second stage of sporulation, thereby preventing the production of spores (29). Sporulation of *B. subtilis* is a complex process involving hundreds of genes, directly or indirectly (30, 31). The activation of the sporulation process will inevitably affect its own anabolism and catabolism. According to our prior research (21), the utilization of the *sigF* locus seems to positively impact the enhancement of MccJ25 production. When employing a multicopy expression strategy to produce MccJ25 in *B. subtilis*, the insertion of the *mcjABCD* gene cluster into the *sigF* gene locus significantly improved MccJ25 production. However, we did not observe similar results when conducting the same study on microcin Y (MccY), which shares the same lasso-like structure as MccJ25. This observation has generated our interest in examining the potential impact of sporulation on the biosynthesis of MccJ25 in *B. subtilis*.

In this study, we investigated the effect of three peptidases (Fig. 1b), PepA, PepF, and PepT, on the heterologous production of MccJ25 by *B. subtilis* and found that PepF could degrade the MccJ25 precursor peptide, thereby reducing the production of mature MccJ25. The absence of PepF enhances the stability of MccJ25 in *B. subtilis*, prolonging the persistence of MccJ25 in the expression products. In addition, we explored the effect of sporulation on the production of MccJ25 in *B. subtilis* and found that the intensity of sporulation was negatively correlated with the expression level of MccJ25 and that preventing sporulation was beneficial for increasing the production of MccJ25 in *B. subtilis*. This study further refined the engineering of *B. subtilis* to enhance the production of MccJ25. The optimizations led to increased deposition of MccJ25 in the cell growth medium and extended its half-life (the period after peak production), thus making it viable for large-scale manufacturing by *B. subtilis*.

## RESULTS

### Identification of key peptidases affecting MccJ25 expression

**Fig 2:**
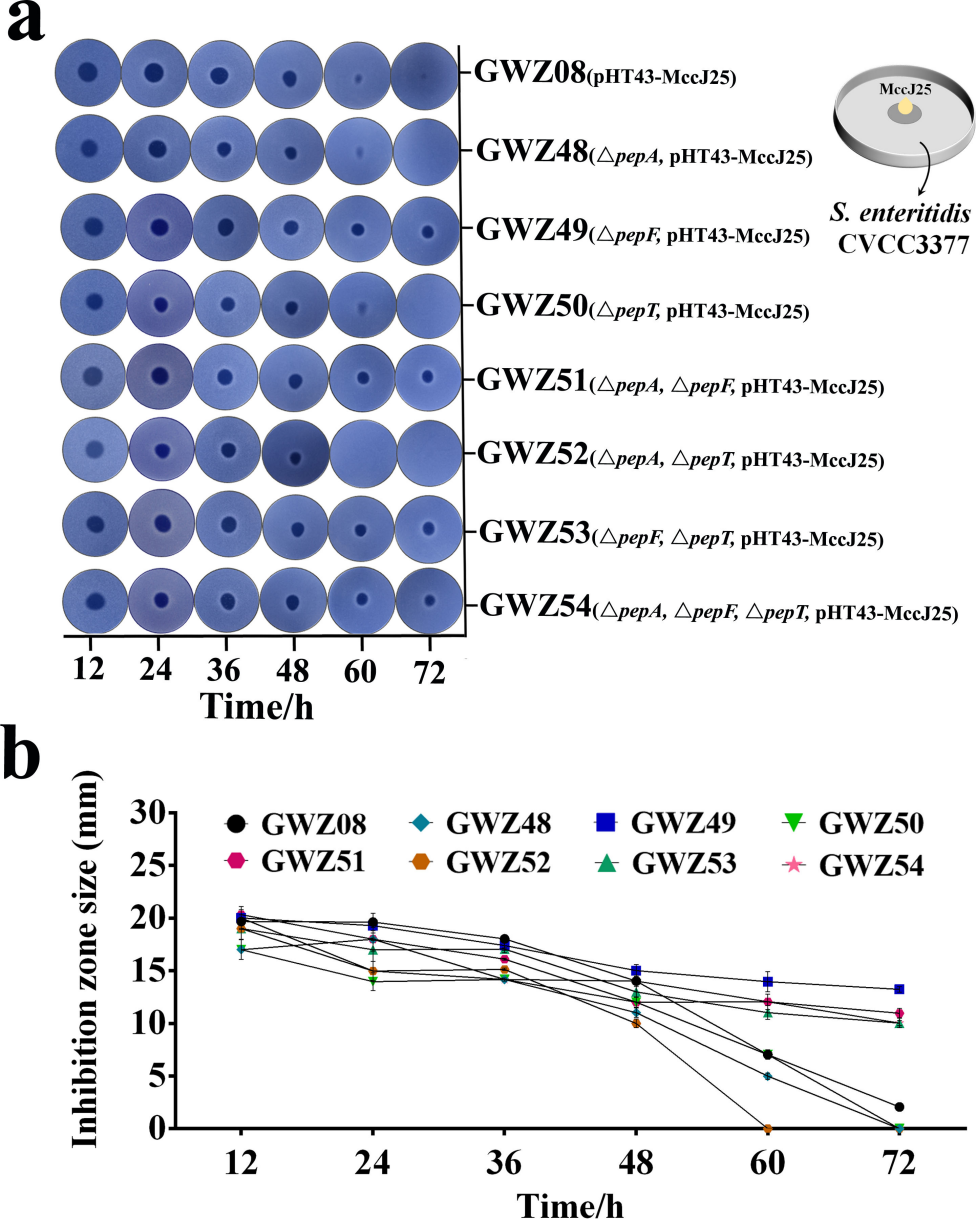
Antibacterial activity of MccJ25-producing *B. subtilis* engineered strains. (a) Spot-on-lawn assay for evaluating the antibacterial activity of the engineered *B. subtilis* WB800N strains GWZ08 (pHT43-MccJ25), GWZ48 (Δ*pepA*, pHT43-MccJ25), GWZ49 (Δ*pepF*, pHT43-MccJ25), GWZ50 (Δ*pepT*, pHT43-MccJ25), GWZ51 (Δ*pepA*, Δ*pepF*, pHT43-MccJ25), GWZ52 (Δ*pepA*, Δ*pepT*, pHT43-MccJ25), GWZ53 (Δ*pepF*, Δ*pepT*, pHT43-MccJ25), and GWZ54 (Δ*pepA*, Δ*pepF*, Δ*pepT*, pHT43-MccJ25) at different times. Activity was measured with the bacterial supernatants, and *S. enteritidis* CVCC3377 was used as the indicator bacteria. (b) The size of the inhibition zone in the supernatant of *B. subtilis* engineered strains GWZ08, GWZ48, GWZ49, GWZ50, GWZ51, GWZ52, GWZ53, and GWZ54 at different times.

Seven engineered strains, namely, GWZ41 (Δ*pepA*), GWZ42 (Δ*pepF*), GWZ43 (Δ*pepT*), GWZ44 (Δ*pepA*, Δ*pepF*), GWZ45 (Δ*pepA*, Δ*pepT*), GWZ46 (Δ*pepF*, Δ*pepT*), and GWZ47 (Δ*pepA*, Δ*pepF*, Δ*pepT*), were constructed, and the pHT43-MccJ25 plasmid was introduced into these mutants and their MccJ25 production levels were analyzed. As shown in the results of the spot-on-lawn assay (Fig. 2a), when MccJ25 was expressed in these engineered strains for more than 48 hours, the amount of MccJ25 in the culture supernatants was different. After 60 hours of expression, the culture supernatants of the GWZ08 (pHT43-MccJ25), GWZ48 (Δ*pepA*, pHT43-MccJ25), GWZ50 (Δ*pepT*, pHT43-MccJ25), and GWZ52 (Δ*pepA*, Δ*pepT*, pHT43-MccJ25) strains showed almost no antibacterial activity, while the culture supernatants of the GWZ49 (Δ*pepF*, pHT43-MccJ25), GWZ51 (Δ*pepA*, Δ*pepF*, pHT43-MccJ25), GWZ53 (Δ*pepF*, Δ*pepT*, pHT43-MccJ25), and GWZ54 (Δ*pepA*, Δ*pepF*, Δ*pepT*, pHT43-MccJ25) strains still had strong antibacterial activity (Fig. 2a). We found that at the late stage of MccJ25 expression, the engineered strains corresponding to the culture supernatants with strong antibacterial activity had a common feature, that is, they lacked the *pepF* gene. In addition, the results of the time-dependent inhibition zone size curves (Fig. 2b) showed that the GWZ49 strain (with single-*pepF* gene deletion) was most conducive to the expression and deposition of MccJ25, and the GWZ52 strain (Δ*pepA* and Δ*pepT*) had the worst performance in production.

### Degradation of mature MccJ25 by peptidases

**Fig 3:**
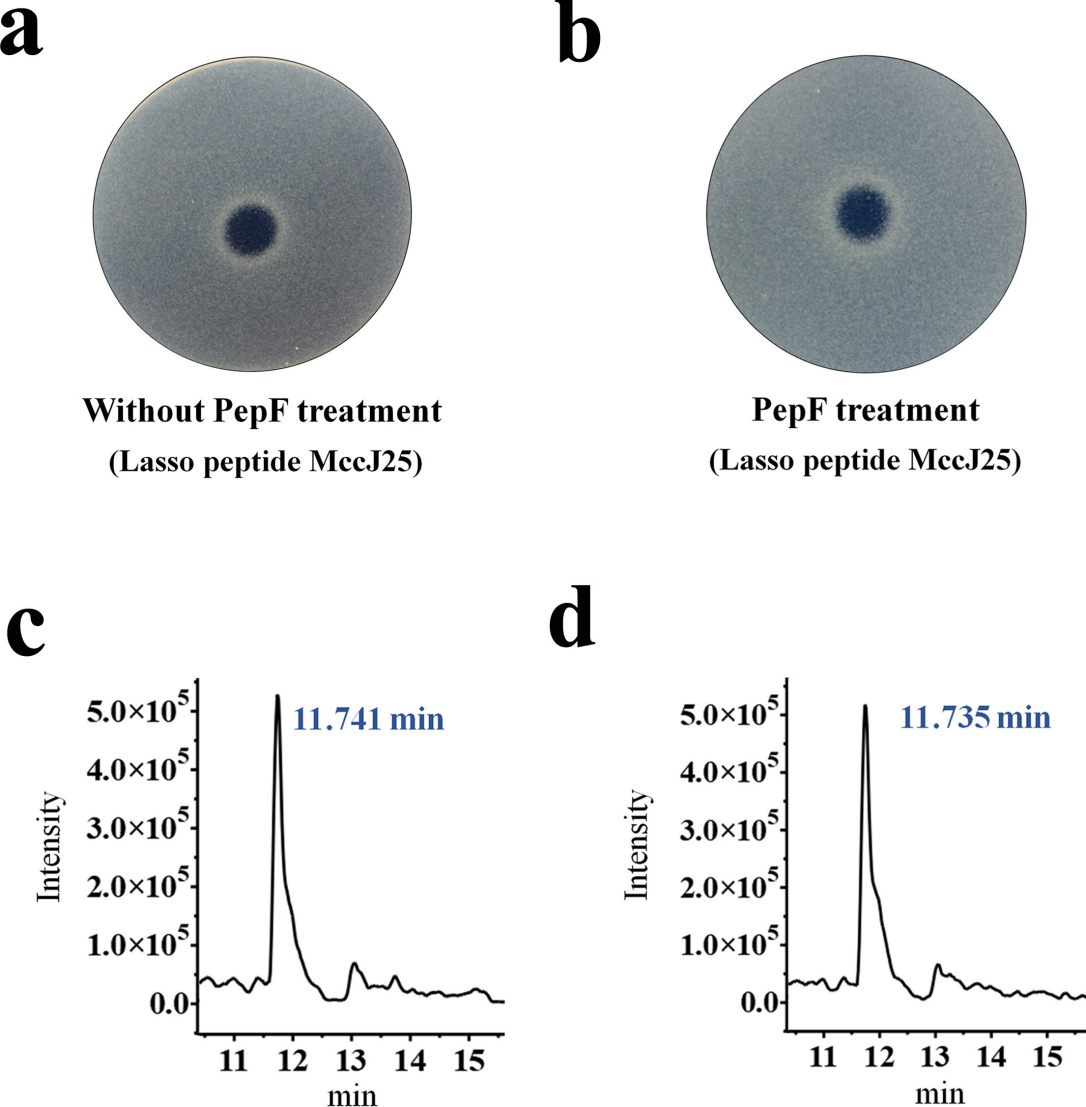
Degradation assay of the MccJ25 lasso peptide by peptidase PepF. (a) Spot-on-lawn assay for evaluating the antibacterial activity of the non-peptidase-treated MccJ25 lasso peptide. Determination of antibacterial activity using *S. enteritidis* CVCC3377 as indicator bacteria. (b) Spot-on-lawn assay for evaluating the antibacterial activity of the MccJ25 lasso peptide after peptidase PepF degradation for 8 hours at 37°C. (c) UPLC-QqQ-MS spectrum identification of the non-peptidase-treated MccJ25 lasso peptide and corresponds to the peak at 11.741 minutes; the *x*-axis represents the retention time, and the *y*-axis represents the relative intensity. (d) UPLC-QqQ-MS spectrum identification of the MccJ25 lasso peptide after 8-h degradation by peptidase PepF and corresponds to the peak at 11.735 minutes.

To examine the degradation effect of these three peptidases on the lasso peptide MccJ25, we performed the enzymatic (proteolytic treatment) reaction of MccJ25 with each peptidase enzyme for 8 hours at 37°C and measured the antibacterial activity and concentration of MccJ25 in the incubation products. Figure 3 shows the results for PepF; panels a and b show that the antibacterial activity of MccJ25 was unchanged after treatment with or without PepF, and panels c and d show that the concentration of MccJ25 was also unchanged. The results indicate that PepF is incapable of degrading the mature lasso peptide MccJ25. Additionally, PepA and PepT exhibit degradation results for the MccJ25 mature peptide, consistent with that of PepF (data not shown). After treatment with these three peptidases, both the antibacterial activity and concentration of mature MccJ25 remained unchanged, indicating that none of these peptidases can degrade mature MccJ25. Here, we determined that the antibacterial activity and concentration of mature MccJ25 in supernatants from cultured *B. subtilis* strains were not affected by the peptidases mentioned above.

### Degradation of the MccJ25 precursor peptide by peptidases

**Fig 4:**
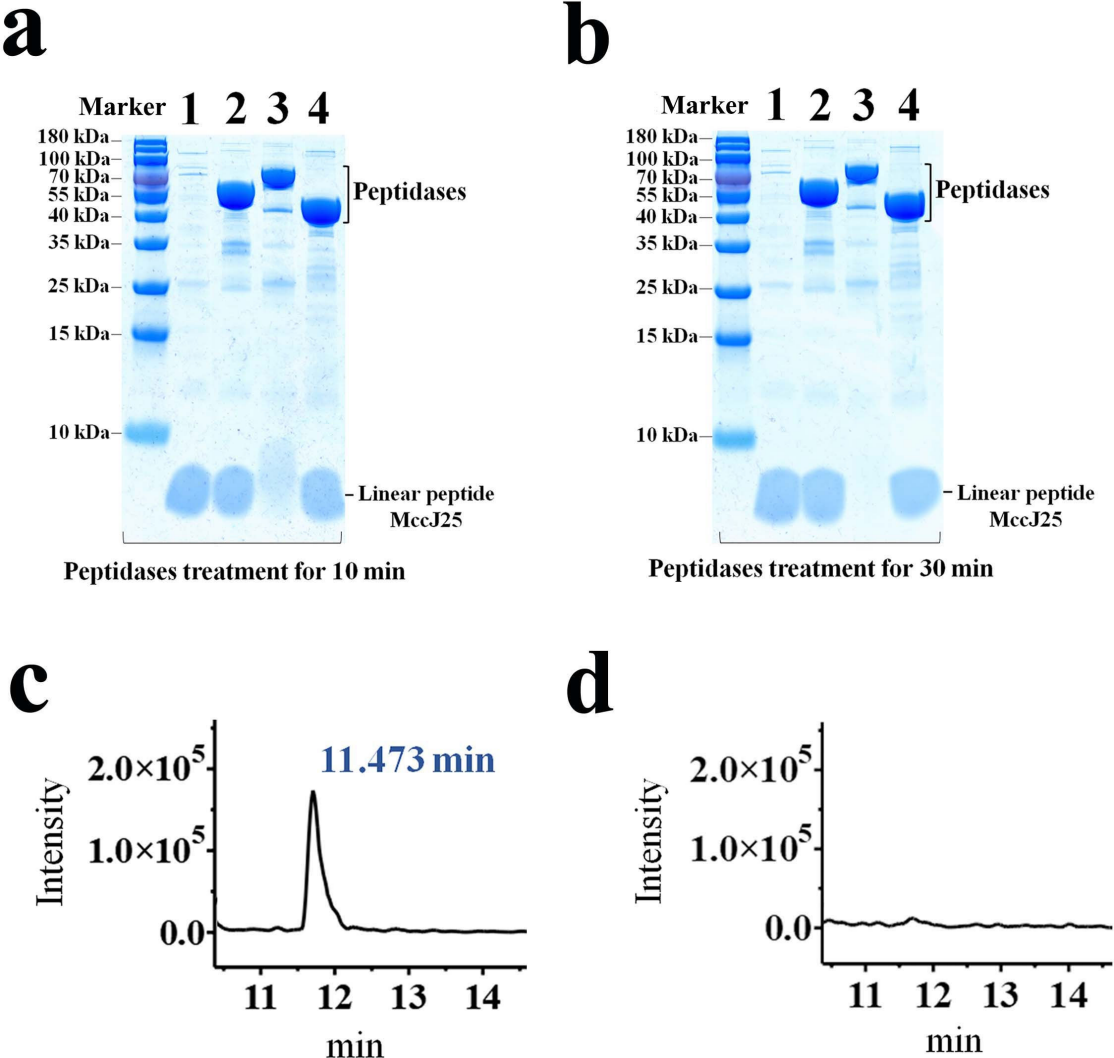
Degradation assay of the MccJ25 linear peptide by peptidases PepA, PepF, and PepT. (a) SDS-PAGE analysis of the MccJ25 linear peptide after 10-min degradation by peptidases. Lane 1, expression product of the BL21O (BL21, pET28a) strain + linear peptide MccJ25 (2 µg); lane 2, PepA + linear peptide MccJ25 (2 µg); lane 3, PepF + linear peptide MccJ25 (2 µg); lane 4, PepT + linear peptide MccJ25 (2 µg). (b) SDS-PAGE analysis of the MccJ25 linear peptide for 30-min degradation. The page lanes are the same as panel a. (c) UPLC-QqQ-MS spectrum identification of the non-peptidase-treated MccJ25 linear peptide and corresponds to the peak at 11.473 minutes; the *x*-axis represents the retention time, and the *y-*axis represents the relative intensity. (d) UPLC-QqQ-MS spectrum identification of the MccJ25 linear peptide after 30-min degradation by peptidase PepF.

To investigate whether *B. subtilis* peptidases play an inhibitory role in the maturation of MccJ25, we synthesized a linear peptide chain consisting of 21 amino acid residues for mimicking the MccJ25 precursor peptide. The linear peptide was incubated with PepA, PepF, and PepT, and the effects of degradation of these three peptidases on the MccJ25 precursor peptide were determined. SDS‒PAGE results showed that neither PepA nor PepT could hydrolyze the MccJ25 linear peptide in the incubation mixture, while PepF could degrade most of the linear peptide within 10 minutes and all of it within 30 minutes (Fig. 4a and b). In addition, we performed UPLC-QqQ mass spectrometry analysis of the incubation mixture and found that compared with the control group, the peak corresponding to the MccJ25 linear peptide in the incubation mixture of the PepF treatment group disappeared, further confirming that the MccJ25 linear peptide was completely degraded by PepF (Fig. 4c and d). These results suggest that PepF in *B. subtilis* can reduce the production of mature MccJ25 by hydrolyzing the MccJ25 precursor peptide.

Furthermore, we employed L-leucine-p-nitroanilide as a colorimetric substrate to assess their aminopeptidase activity. Following the experimental methods described previously, we expressed and purified these three enzyme proteins and conducted enzyme activity analysis. As observed in the results shown in Fig. S2b, the PepA group and PepF group did not exhibit any color reaction, while the PepT group showed a visible color reaction. These results indicated that *B. subtilis* PepT is an aminopeptidase, while PepA and PepF are not.

### Effects of peptidases on MccJ25 production in *B. subtilis*

**Fig 5:**
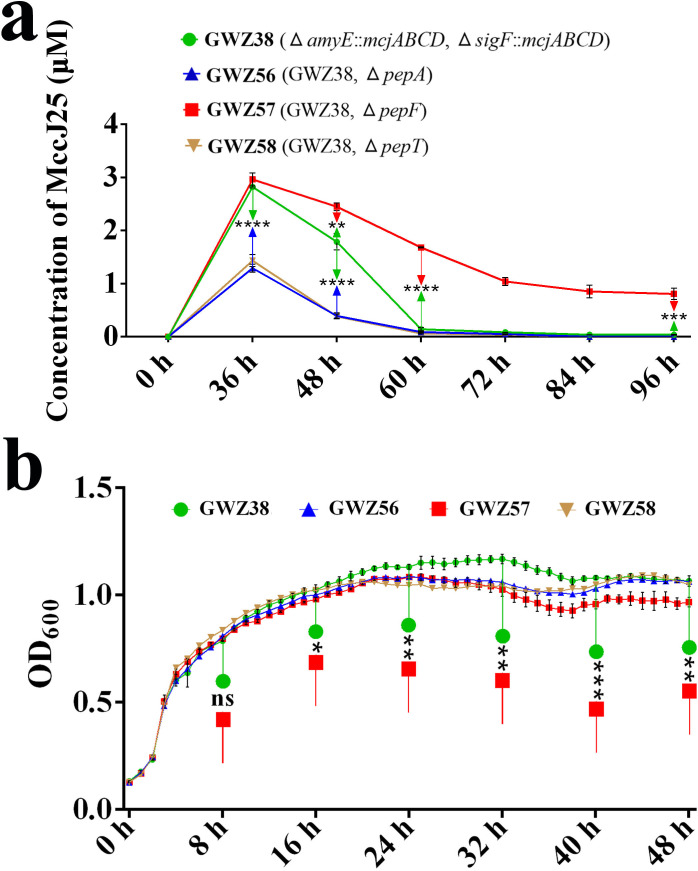
Effects of peptidase gene deletion on maintenance of MccJ25 production and cell growth performance in the *B. subtilis* WB800N strain. (a) The MccJ25 expression levels of GWZ38 (WB800N, Δ*amyE*::MccJ25 and Δ*sigF*::MccJ25), GWZ56 (GWZ38, Δ*pepA*), GWZ57 (GWZ38, Δ*pepF*), and GWZ58 (GWZ38, Δ*pepT*) strains in the shake-flask culture within 96 hours. (b) Cell growth performance of the GWZ38, GWZ56, GWZ57, and GWZ58 strains. All data were expressed as the average of three independent experiments with standard deviations. Data represent the means ± SD, *n* = 3; * means *P* < 0.05; ** means *P* < 0.01; ns means no significant difference.

To investigate the effects of three peptidases, PepA, PepF, and PepT, on MccJ25 expression level in *B. subtilis*, we constructed three peptidase deletion strains, GWZ56 (GWZ38, Δ*pepA*), GWZ57 (GWZ38, Δ*pepF*), and GWZ58 (GWZ38, Δ*pepT*), based on the GWZ38 strain. The GWZ38 (WB800N, Δ*amyE*::MccJ25 and Δ*sigF*::MccJ25) strain integrates the *mcjABCD* gene cluster into both the *amyE* and *sigF* loci, enabling efficient expression of MccJ25 without the need for inducers or antibiotics. We measured the concentration of MccJ25 in the supernatants of these peptidase-deficient strains at different time points within 96 hours. The results showed that, compared with the original GWZ38 strain, the GWZ38 strain with deletion of the *pepA* and *pepT* genes had a significantly lower peak expression of MccJ25. When the expression time for these three strains (GWZ38, GWZ56, and GWZ58) reached 60 hours, the MccJ25 in the culture supernatant was almost completely consumed (0.0632–0.1431 µM, Fig. 5a). However, the peak value of MccJ25 production level in *pepF* gene-deleted strains was higher than that in the original strain, and there was still a high concentration of MccJ25 (1.6817 µM) in the culture supernatant of the *pepF* gene-deleted strains after an expression time of 60 hours (Fig. 5a). These results indicate that the peptidase PepF is a key factor affecting the expression of MccJ25 in *B. subtilis* and that deletion of the *pepF* gene can prolong the half-life of MccJ25 in the culture supernatant. To further verify this phenotype, we constructed the pHT43-PepF plasmid for complementation of the PepF-deficient strain, and the results showed that the expression level of MccJ25 was lower (1.3685 µM vs 2.9604 µM) and that the MccJ25 half-life was shorter in the PepF-overexpressing strain than in the PepF-deficient strain (Fig. S3).

To explore the reason for the lower expression level of MccJ25 in peptidase PepA- and PepT-deficient strains, we evaluated the cell growth performance of all the peptidase-deficient strains in this study. The results showed that the bacterial colony density in the late logarithmic growth phase of cell growth of the single-deletion strain for any one of the three peptidases was decreased compared to that of the original strain (Fig. 5b; Fig. S4a and b). Moreover, this reduction in cell growth performance had an additive effect after the deletion of multiple peptidase genes, and strains GWZ47 and GWZ54, which lacked all three peptidases, exhibited the lowest cell colony density during the late logarithmic growth phase (Fig. S4a and c). These results indicate that the reason for the decreased expression of MccJ25 in the GWZ56 and GWZ58 strains is probably that the deletion of the peptidase genes *pepA* and *pepT* reduced the cell growth colony density of *B. subtilis*. The same result is also observed in the GWZ57 strain, but because the positive effect of *pepF* gene deletion masked the negative effect, a net positive effect was observed.

### Transcription level of the *mcjABCD* gene in the *pepF* gene deletion strain

**Fig 6:**
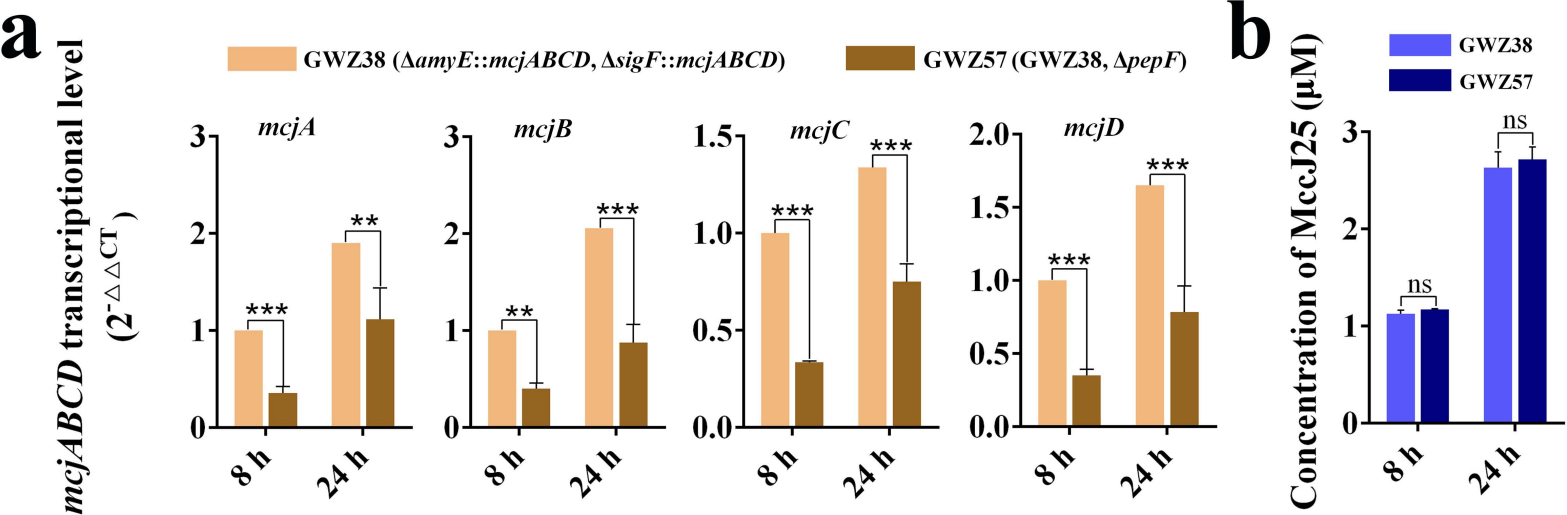
Transcriptional level of *mcjABCD* genes and production of MccJ25 in *B. subtilis* strains with deletion of the *pepF* gene. (a) Comparison of the transcriptional level of genes *mcjA*, *mcjB*, *mcjC,* and *mcjD* in GWZ38 (WB800N, Δ*amyE::mcjABCD*, Δ*sigF::mcjABCD*) and GWZ57 (GWZ38, Δ*pepF*) strains at 8 hours and 24 hours. (b) Comparison of the MccJ25 expression level in GWZ38 and GWZ57 strains at 8 hours and 24 hours. Data represent the means ± SD; *n* = 3; * means *P* < 0.05; ** means *P* < 0.01; ns means no significant difference.

According to the results of cell growth performance analysis, we speculated that deletion of the *pepF* gene impairs the ability of *B. subtilis* cells to utilize nutrients, thereby negatively affecting the expression of MccJ25. Therefore, we investigated the transcript levels of the MccJ25 biosynthetic gene cluster in *pepF* gene deletion strains. The transcript levels of the *mcjABCD* genes were determined by the quantitative real-time polymerase chain reaction. The results showed that the transcript levels of the four genes in the GWZ57 strain were significantly lower than those in the GWZ38 strain (*P* < 0.05), and this result was consistent whether the strain was expressed for 8 hours or 24 hours (Fig. 6a). However, production of MccJ25 in the GWZ57 strain did not decrease compared with that in the GWZ38 strain, showing a tendency to increase instead (Fig. 6b). These results indicate that the intact *pepF* gene helps *B. subtilis* maintain good cell growth performance, thereby stabilizing the expression of MccJ25.

### Correlation analysis between MccJ25 production and the sporulation process in *B. subtilis*

**Fig 7:**
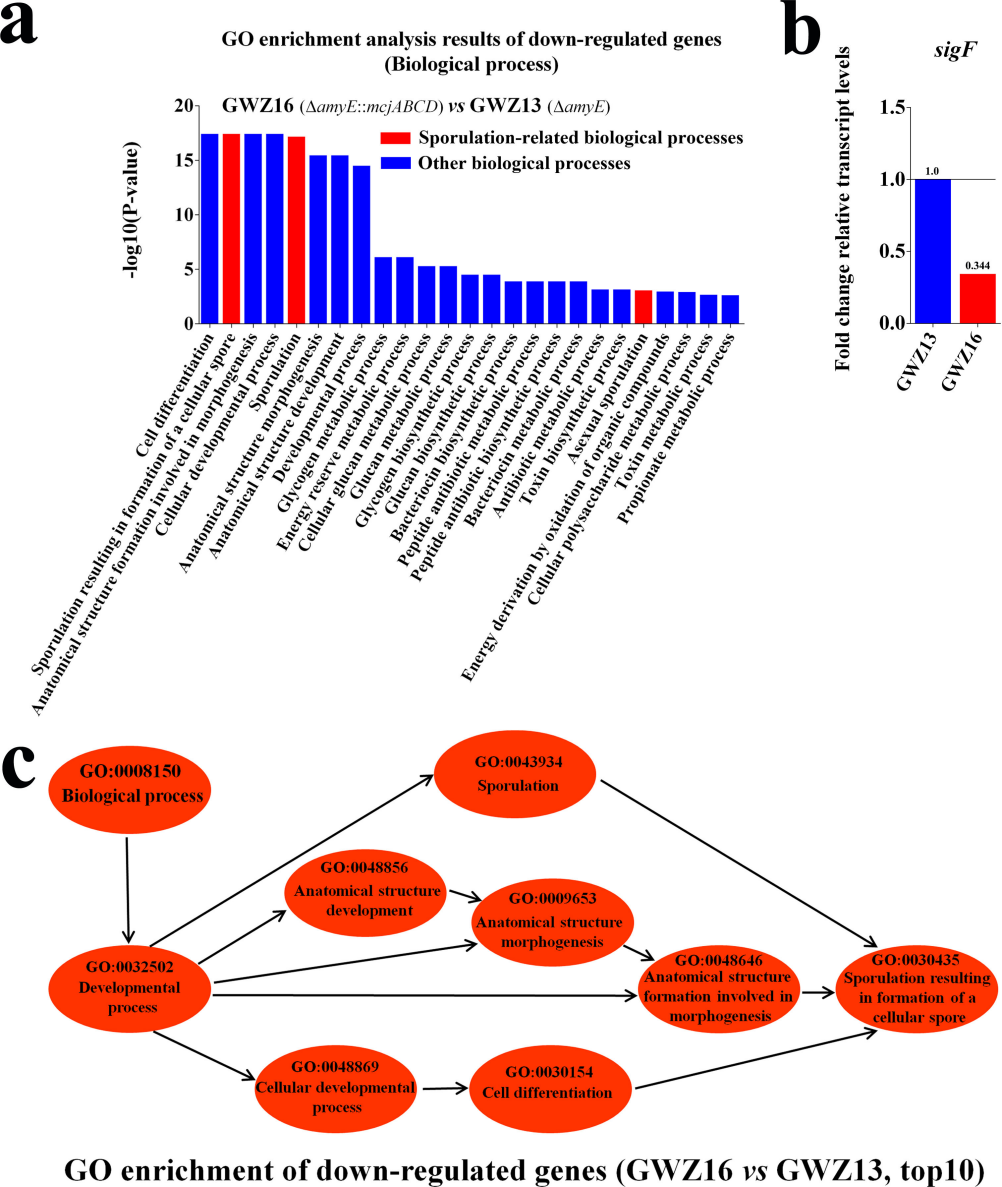
Transcriptome analysis of the GWZ13 (WB800N, Δ*amyE*) and GWZ16 (WB800N, Δ*amyE::mcjABCD*) strains. (a) GO enrichment results of top 25 down-regulated biological processes. (b) Comparison of *sigF* gene transcription levels between GWZ13 and GWZ16 strains. (c) GO directed acyclic graph of top 10 down-regulated biological processes.

In order to explore the relationship between MccJ25 synthesis and spore formation in *B. subtilis*, transcriptome analysis of the engineered strains GWZ13 (WB800N, Δ*amyE*) and GWZ16 (WB800N, Δ*amyE::mcjABCD*) was carried out in this study. The results of GO enrichment analysis showed that the expression of MccJ25 in *B. subtilis* caused downregulation of the gene transcription levels related to the biological process of sporulation (Fig. 7a; Table S3). Compared with the GWZ13 strain, the GWZ16 strain exhibited threefold downregulation of the *sigF* gene transcription level (Fig. 7b; Table S4), and downregulation of cell biological processes was observed during the spore formation process (Fig. 7c; Table S5). These results indicate that the expression of MccJ25 in *B. subtilis* reduces its sporulation biological activity.

### Effects of *B. subtilis* sporulation on the expression level of MccJ25

**Fig 8:**
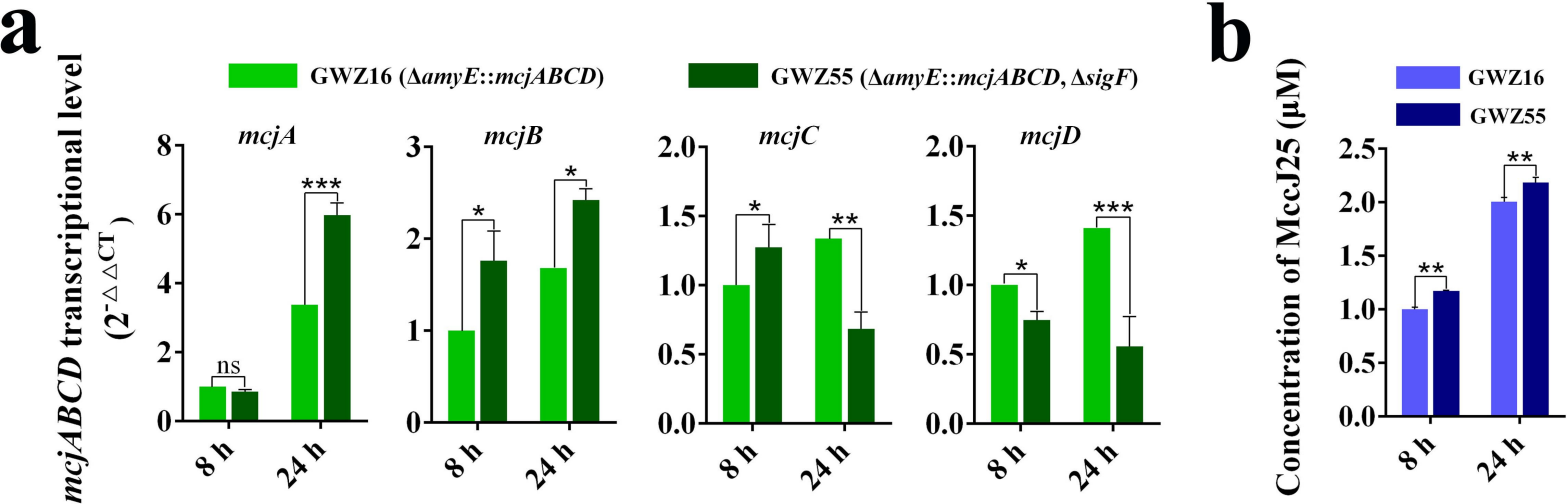
Transcriptional level of *mcjABCD* genes and production of MccJ25 in *B. subtilis* strains with deletion of the *sigF* gene. (a) Comparison of the transcriptional level of genes *mcjA*, *mcjB*, *mcjC,* and *mcjD* in GWZ16 (WB800N, Δ*amyE::mcjABCD*) and GWZ55 (GWZ16, *sigF*) strains at 8 hours and 24 hours. (b) Comparison of MccJ25 expression level in GWZ16 and GWZ55 strains at 8 hours and 24 hours. Data represent the means ± SD; *n* = 3; * means *P* < 0.05; ** means *P* < 0.01; ns means no significant difference.

In this study, two engineered strains, GWZ16 (WB800N, Δ*amyE::mcjABCD*) and GWZ55 (GWZ16, Δ*sigF*), were used to explore the effect of *B. subtilis* sporulation on MccJ25 expression performance. We compared the transcript levels of the *mcjABCD* genes and the concentration of MccJ25 in the two strains GWZ16 and GWZ55. The results showed that after 24 hours of expression, the transcript level of the *mcjA* gene in the GWZ55 strain was higher than that in the GWZ16 strain (Fig. 8a), and the MccJ25 yield of the GWZ55 strain (1.17 and 2.18 µM) was higher than that of the GWZ16 strain (1.01 and 1.99 µM) regardless of whether it was expressed for 8 hours or 24 hours (Fig. 8b). These results suggest that defects in sporulation are advantageous for the production of MccJ25 in *B. subtilis*.

**Fig 9:**
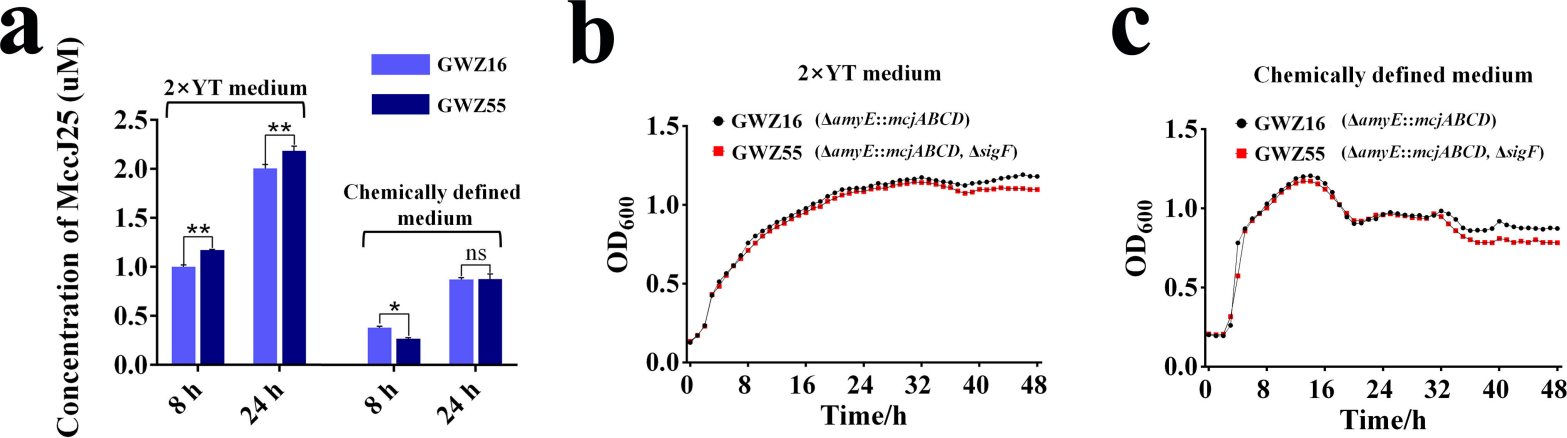
Comparison of the MccJ25 expression level and cell growth performance of *B. subtilis* strains with deletion of the *sigF* gene in different culture media. (a) Expression levels of MccJ25 in *B. subtilis* GWZ16 (WB800N, Δ*amyE::mcjABCD*) and GWZ55 (GWZ16, *sigF*) strains in different culture media (2× YT medium and chemically defined medium) at 8 hours and 24 hours. Data represent the means ± SD; *n* = 3; * means *P* < 0.05; ** means *P* < 0.01; ns means no significant difference. (b) Cell growth performance of GWZ16 and GWZ55 strains in the 2× YT culture medium. (c) Cell growth performance of GWZ16 and GWZ55 strains in the chemically defined medium. All data were expressed as the average of three independent experiments with standard deviations.

Additionally, to further verify this conclusion, we prepared the chemically defined medium and assayed the concentration of MccJ25 in the supernatants of the GWZ16 and GWZ55 strains. We found that under nutrient-limited growth conditions, *B. subtilis* sporulation had no effect on its cell density and the final expression of MccJ25 (Fig. 9), and the expression level of MccJ25 in the GWZ55 strain was even lower than that in the GWZ16 strain after 8 hours of expression (Fig. 9a). In the 2× YT medium (Fig. 9b) and chemically defined medium (Fig. 9c), there were no differences in the growth performance of the GWZ55 and GWZ16 strains. Based on these results, it is suggested that the disruption of the sporulation capacity of MccJ25-producing engineered strains should be taken into consideration when cultivating them in nutrient-rich environmental conditions.

## DISCUSSION

Our previous research established a method for the heterologous production of MccJ25 in *B. subtilis* and constructed the engineered strain GWZ38 for the production of MccJ25 (21). However, one phenomenon caught our attention, that is, the production of MccJ25 peaked after 36 hours of expression and then decreased rapidly over time, whereas the expression of the other microcin, MccY, did not exhibit this phenomenon. This indicates that there are unknown factors affecting the deposition of MccJ25 in the expression product during the production of MccJ25 by *B. subtilis*. They reduce the production level of MccJ25 and limit the application of engineered bacteria. Research has revealed that the three nonspecific oligopeptidases PepA, PepB, and PepN in *E. coli* are capable of processing microcin C. The existence of these peptidases hinders the production and accumulation of microcin C (32). In view of the related reports on this microcin, we speculate that the peptidases in *B. subtilis* are involved in the degradation of MccJ25.

In this study, to determine whether peptidases in *B. subtilis* are involved in the degradation of MccJ25, we investigated the MccJ25 production levels in *B. subtilis* WB800N strains with singly, doubly, and triply disrupted peptidase genes (*pepA*, *pepF,* and *pepT*). The results indicate that the oligopeptidase PepF is probably involved in the reduction of MccJ25 production in *B. subtilis*. Since there is no research on the degradation of MccJ25 by *B. subtilis* peptidases, the specific mechanism needs to be further studied.

The MccJ25 consists of 21 amino acid residues, including an eight-residue cycle and a thirteen-residue tail (3), and the slipknot-like structure formed by tail threading through the macrolactam ring strengthens the stability of mature MccJ25 (33). Although previous studies have noted that the lasso peptide MccJ25 exhibits good resistance under protease activity (1), the ability of degradation of MccJ25 by *B. subtilis* peptidases PepA, PepF, and PepT has not been reported. This study explored the degradation effects of these three peptidases on the MccJ25 mature peptide and MccJ25 linear peptide. The results showed that none of the three peptidases could degrade mature MccJ25, and only PepF could degrade the MccJ25 precursor peptide. This suggests that PepF in *B. subtilis* can reduce the production of mature MccJ25 by hydrolyzing the MccJ25 precursor peptide. Therefore, deletion of the *pepF* gene should increase the production of MccJ25 in *B. subtilis*, but in fact, there was no dramatic increase observed in the MccJ25 expression level. This is due to the deletion of the peptidase genes, which resulted in a reduction in the cell growth colony density of the host strain and a decrease in the global transcriptional activity of the MccJ25-encoding gene clusters. Our previous research has found that the expression level of MccJ25 in *B. subtilis* is positively correlated with the overall transcriptional activity of the *mcj* cluster (21).

We believe that identification of the peptidase species that hydrolyze the MccJ25 precursor peptide in *B. subtilis* is important for the development of engineered bacteria. In *B. subtilis*, PepA and PepT are bioinformatically predicted to function as aminopeptidases, and PepF is predicted to function as an oligoendopeptidase. In this study, *B. subtilis* PepT was identified as an aminopeptidase, while PepA and PepF were not, which is inconsistent with the results reported in previous studies (25, 27). The homology between *B. subtilis* PepA and *E. coli* PepA is relatively low, which could be one of the reasons for the lack of aminopeptidase activity in *B. subtilis* PepA. Peptidases are classified as exopeptidases or endopeptidases according to the position of peptide bond cleavage. Exopeptidases are classified as aminopeptidases or carboxypeptidases according to their site of action (N or C terminus, respectively), and they act only near the ends of polypeptide chains (22). Endopeptidases preferentially act on peptide bonds away from the N and C termini within the polypeptide chain (22). Therefore, *B. subtilis* endopeptidases are more likely to have the ability to degrade the MccJ25 precursor peptide than *B. subtilis* exopeptidases.

In the previous study, we found that when the MccJ25 biosynthetic gene cluster was simultaneously introduced into the gene loci of *amyE* and *sigF*, the yield of MccJ25 was significantly higher than the sum of the yields when the two gene loci expressed MccJ25 separately (21). We speculate that this is due to the disruption of the *sigF* gene, resulting in impaired sporulation function of the host strain, which in turn affects the expression level of MccJ25. As an excellent expression system, *B. subtilis* is often used to produce recombinant proteins (34). However, the expression of heterologous proteins often causes changes in various functions of *B. subtilis*, and these changes often affect the yield of target proteins in turn. The sporulation process of *B. subtilis* is strongly affected by various environmental conditions and intracellular protein expression (35). In addition, research has indicated that the overexpression of PepF can inhibit the initiation of spore formation in *B. subtilis* (26), suggesting a potential association between sporulation and this peptidase, as well as the synthesis of MccJ25. In this study, we discovered that the expression of MccJ25 in *B. subtilis* inhibits the biological process of sporulation, while the reduction in the sporulation ability of the host strain increases the production of MccJ25 via a feedback effect. Nevertheless, despite the inhibitory effects of both the peptidase PepF and the sporulation process on MccJ25 production in *B. subtilis*, we currently lack direct evidence demonstrating that the interaction between PepF and sporulation can impact MccJ25 production.

The sporulation process of *B. subtilis* proceeds through a series of complex morphological stages, wherein sigma factor F controls genes required for the early stages of spore development (29, 36), and the deletion of the *sigF* gene leads to the arrest of sporulation in *B. subtilis*. During this study, it was observed that the diminished sporulation function of *B. subtilis* led to a significant increase in MccJ25 production, primarily attributed to the upregulation of the *mcjA* gene’s transcription level. Previous studies have shown that the production level of MccJ25 in *B. subtilis* mainly depends on the transcription level of the *mcjA* gene (21). Although the lack of sporulation ability of the host strain negatively affected the transcription levels of the MccJ25 posttranslational modifier gene *mcjC* and self-immunity-related gene *mcjD*, the upregulation in the transcription level of the precursor peptide-coding gene *mcjA* resulted in a significant increase in the final MccJ25 yield.

It is important to note that the effect of the sporulation process of *B. subtilis* on MccJ25 production becomes more evident in nutrient-rich media. *B. subtilis* initiates its sporulation process through a quorum-sensing system, which takes place in densely populated bacterial communities and only when the cells are under starvation stress (37). Therefore, the reason why the spore-deficient strain has a higher expression of MccJ25 than the original strain in the nutrient-rich medium is that the spore formation process of the original strain competes for and utilizes a portion of the available nutrient resources. A similar phenomenon was not observed in the chemically defined medium, possibly because the low-nutrient medium is not conducive to MccJ25 expression by *B. subtilis*, resulting in no significant difference in its production.

### Conclusions

In this study, we have provided evidence indicating that both peptidase PepF and sporulation are significant factors that limit the expression of MccJ25 in *B. subtilis*. Deleting the *pepF* and *sigF* genes has the effect of increasing MccJ25 production and prolonging its half-life. At the same time, we determined that the *B. subtilis* peptidase PepF influences the production level of MccJ25 through two opposite aspects: one involves the hydrolysis of the MccJ25 precursor peptide to inhibit its production and the other involves maintaining a high cell growth colony density to stabilize MccJ25 expression. The final yield of MccJ25 in *B. subtilis* is determined by the collective influence of the aforementioned two factors. In addition, when cultivating excellent MccJ25 production engineering strains, destroying the sporulation ability of *B. subtilis* should be considered. Finally, the GWZ42 strain constructed in this study is more conducive to the deposition of MccJ25 in the culture supernatant of *B. subtilis* in the late stages of expression when producing MccJ25 from the plasmid. The developed GWZ57 strain has better deposition ability for MccJ25 than GWZ38, which greatly alleviates the rapid decline in the expression level of MccJ25 after 36 hours. Although the level of MccJ25 in the culture products of the *pepF* deletion strain still shows a slowly declining trend, it provides a research basis for continuing to optimize the production of MccJ25 in *B. subtilis* in the future. Based on the results of this work, there are two potential solutions that can be used to further overcome the effects of depletion of MccJ25 in *B. subtilis* culture supernatants. First, to develop a homolog of the peptidase PepF that cannot degrade the MccJ25 linear peptide to compensate for the reduced utilization of nutrients in the culture environment by strains caused by the absence of *pepF*; second, to explore other unknown key factors that affect MccJ25 deposition to further avoid degradation of the target protein in late stages of cell expression. These efforts will be of great significance for realizing large-scale and low-cost production of microcins in probiotics.

## MATERIALS AND METHODS

### Plasmids, strains, and culture medium

The plasmids and strains used in this study are listed in Table S1. The plasmid pHT43-MccJ25 and strains GWZ08 (WB800N, pHT43-MccJ25), GWZ13 (WB800N, Δ*amyE*), GWZ16 (WB800N, Δ*amyE::mcjABCD*), and GWZ38 (WB800N, Δ*amyE::mcjABCD*, Δ*sigF::mcjABCD*) were derived from our previous research (21). The construction of the recombinant plasmids pET28a-PepA, pET28a-PepF, and pET28a-PepT was achieved by linking the linearized pET28a(+) plasmid with the three peptidase genes by an In-Fusion cloning kit (Clontech), and a similar method was employed to obtain the recombinant plasmid pHT43-PepF. The strains *Salmonella enteritidis* CVCC3377, *E. coli* DH5α, and *E. coli* BL21(DE3) were grown in LB medium (5 g/L yeast extract, 10 g/L tryptone, and 5 g/L NaCl) at 37°C. *B. subtilis* strains were grown in 2× YT medium (10 g/L yeast extract, 16 g/L tryptone, and 5 g/L NaCl) or chemically defined medium (sucrose, 5 g/L; K_2_HPO_4_, 3 g/L; NH_4_Cl, 1 g/L; NaH_2_PO_4_, 1 g/L; MgSO_4_ · 7H_2_O, 200 mg/L; KCl, 125 mg/L; CaCl_2_, 20 mg/L; FeSO_4_ · 7H_2_O, 2 mg/L) at 37°C. Tryptone and yeast extract were obtained from Thermo Fisher (USA). Antibiotics and inducers such as spectinomycin (100 µg/mL), ampicillin (100 µg/mL), kanamycin (30 µg/mL), chloramphenicol (10 µg/mL), isopropyl β-D-thiogalactoside (IPTG, 1.5 mM), and 5-fluorouracil (5-FU; 10 µM) were supplemented appropriately.

### Construction of engineered strains and analysis of cell growth curves

The transformation of *E. coli* was carried out according to the instructions provided by the competent cell manufacturer (Vazyme Biotech Co., Ltd., Nanjing, China). The electroporation method for *B. subtilis* was the same as that described in a previous article (38). The *pepA*, *pepF*, and *pepT* genes of *B. subtilis* were deleted by a homologous recombination method (39), and the operation schematic diagram is shown in Fig. S1a. The transformants were confirmed by PCR and visualized on an agarose gel (Fig. S1b). The primers and DR sequences are listed in Table S2. Subsequently, the cell growth performance of all the engineered strains in this study was determined using the method described in our previous study (21).

### Expression of MccJ25 in *B. subtilis*

*B. subtilis* strains were inoculated individually into 2× YT medium as seed cultures. After shaking overnight, each seed culture was inoculated into fresh 2× YT medium (with an inoculation dose of 10%), while IPTG was added at a final concentration of 1.5 mM to induce the expression of MccJ25. The cultures were then centrifuged at 12,000 × *g* at different time points to harvest the supernatants for subsequent analysis.

### Synthesis and analysis of MccJ25

The MccJ25 linear peptide was synthesized by GENEWIZ (USA), and its amino acid sequence is shown in Fig. 1a. Analysis of the MccJ25 linear peptide was performed by SDS‒PAGE and UPLC-QqQ mass spectrometry. The MccJ25 lasso peptide was obtained by a reverse-phase solid-phase extraction method, as described in previous studies (15). The MccJ25 lasso peptide was examined by antimicrobial activity analysis and UPLC-QqQ mass spectrometry analysis. The antibacterial activity analysis method and UPLC-QqQ mass spectrometry analysis process for MccJ25 have been described in detail in our previous articles (21). A spot-on-lawn assay was conducted to confirm the bactericidal activity of the lasso peptide MccJ25, and the indicator bacterium was *S. enteritidis* CVCC3377. Quantitative analysis of the MccJ25 linear/lasso peptide was performed by ultra-performance liquid chromatography coupled with triple quadrupole mass spectrometry (UPLC-QqQ, Agilent Co., USA) in the positive ion mode. All original images of SDS-PAGE and spot-on-lawn were cropped and brightness adjusted using Fireworks software (Vesion CS 6, Adobe Systems Incorporated). Due to artifacts created during the image processing, there might be a faint line visible in some images (e.g., Fig. 4a).

### Production and characterization of the peptidases PepA, PepF, and PepT

The strains BL21A (BL21-DE3, pET28a-PepA), BL21F (BL21-DE3, pET28a-PepF), and BL21T (BL21-DE3, pET28a-PepT) were used to produce the peptidases PepA, PepF, and PepT, respectively, and BL21O (BL21-DE3, pET28a+) was used as a control strain. All three peptidases were purified by Ni-NTA affinity chromatography, as described in a previous report (40), and the expression of the peptidases was determined by SDS‒PAGE (Fig. S2a). L-leucine-p-nitroanilide was used as the standard chromogenic substrate for aminopeptidases to identify whether these three peptidases are aminopeptidases in *B. subtilis*. The mixture of peptidase (10%) and L-leucine-p- nitroanilide (1 mM) was reacted in Tris-HCl buffer solution at pH = 8.5 and 50°C for 10 minutes. If brown p-nitroanilide was produced, it was determined that the peptidase had aminopeptidase properties (Fig. S2b).

### Transcriptome analysis of *B. subtilis* strains

The recombinant *B. subtilis* strains GWZ13 (WB800N, Δ*amyE*) and GWZ16 (WB800N, Δ*amyE::mcjABCD*) were cultured for 8 hours according to the method mentioned in “Expression of MccJ25 in *B. subtilis*.” The total RNA of these strains was then extracted (TRIzol reagent). The cDNA library was constructed and sequenced on an Illumina NovaSeq 6000 system (41). Differences between GWZ13 and GWZ16 strains were revealed by GO enrichment analysis of biological processes associated with the genes. The data are shown in Tables S3 to S5. Each strain was sequenced three times independently.

### qPCR analysis of *mcj* gene transcription levels

Samples were collected after the strains were cultured for 8 hours and 24 hours, and the sediments obtained from the centrifugation of the bacterial cultures at 12,000 × *g* for 15 minutes were selected to extract total RNA (E.Z.N.A. Bacterial RNA Kit, Omega). Specific oligonucleotides were used as primers to reverse-transcribe the RNA strands of *mcjA*, *mcjB*, *mcjC,* and *mcjD* to cDNA (Takara Bio Inc.), and *rpoB* was used as the reference gene. The expression analysis of these five genes was performed by using the 7300 Real-Time PCR System (Applied Biosystems). The data were analyzed using the 2^∆∆Ct^ method.

### Statistical analysis

The size of the inhibition zone was based on the transverse diameter. The statistical significance of differences was determined using one-way ANOVA in GraphPad Prism (Prism 6) software. Unless specified otherwise, all experiments were performed in triplicate.

## SUPPLEMENTAL MATERIAL

Supplemental material spectrum.03748-23-s0001.docx Tables S1 and S2; Fig. S1 to S4.

Supplemental tables spectrum.03748-23-s0002.xlsx Tables S3 to S5.

ASM does not own the copyrights to Supplemental Material that may be linked to, or accessed through, an article. The authors have granted ASM a non-exclusive, world-wide license to publish the Supplemental Material files. Please contact the corresponding author directly for reuse.
