# Supplementary material for: Influence of PepF peptidase and sporulation on microcin J25 production in Bacillus subtilis
Source: Microbiol Spectr. 2024 May 23;12(7):e03748-23. doi: 10.1128/spectrum.03748-23 (PMC11218540; doi:10.1128/spectrum.03748-23)
Supplement: Supplemental material — Tables S1 and S2; Fig. S1 to S4. [file spectrum.03748-23-s0001.docx]

**Supplementary data**

**Influence of PepF peptidase and sporulation on microcin J25 production in *Bacillus subtilis***

**Authors:** Guangwen Zhang^1#^, Saixiang Feng^1,2,3,4,5#^, Miaomiao Qin^1^, Juan Sun^1^, Yutong Liu^1^, Changqi Luo^1^, Min Lin^1^, Siqi Xu^1^, Ming Liao^1,2,3,4,5,6^, Huiying Fan^1,2,4,5^*, Zhaoping Liang^1^*

^1^College of Veterinary Medicine, South China Agricultural University, Guangzhou 510642, P.R. China. ^2^Key Laboratory of Zoonosis Prevention and Control of Guangdong Province, Guangzhou 510642, P.R. China. ^3^Guangdong Laboratory for Lingnan Modern Agriculture, South China Agricultural University, Guangzhou 510642, P.R. China. ^4^Key Laboratory of Veterinary Vaccine Innovation of the Ministry of Agriculture, Guangzhou 510642, P.R. China. ^5^National and Regional Joint Engineering Laboratory for Medicament of Zoonosis Prevention and Control, Guangzhou 510642, P.R. China. ^6^Institute of Animal Health, Guangdong Academy of Agricultural Sciences, Guangzhou, 510642, P.R. China.

***Corresponding author:**

Zhaoping Liang: Tel/Fax, +86-20-85280718; *E-mail: liangzp1017@126.com

Huiying Fan: Tel/Fax, +86-20-85283309; *E-mail: fanhy@scau.edu.cn

# These authors contributed equally to this work

**Table S1.** Plasmids and strains used in this study.

| **Plasmids** | **Description** | | | **Source/reference** |  |
| --- | --- | --- | --- | --- | --- |
| pHT43 | *Ampr*, *Chlr*, *lacO*, *B. subtilis-E. coli* shuttle expression vector | | | Laboratory stock |  |
| pHT43-MccJ25 | pHT43-P*_43_*–*mcjA*–P*_veg_*–*mcjBCD* | | | Zhang *et al*. (2023) |  |
| pET28a(+) | *Kanr*, for expression of peptidases | | | Laboratory stock |  |
| pET28a-PepA | pET28a(+)-*pepA* | | | This study |  |
| pET28a-PepF | pET28a(+)-*pepF* | | | This study |  |
| pET28a-PepT | pET28a(+)-*pepT* | | | This study |  |
| pHT43-PepF | pHT43-*pepF* | | | This study |  |
| **Strains** |  | | |  |  |
| *E. coli* DH5α | | F^-^, φ80d/*lacZ*ΔM15, Δ(*lacZYA*-*argF*) U169 *recA1 endA1 hsdR*17 | Laboratory stock | | |
| *E. coli* BL21(DE3) | | F-, *ompT*, *hsdS(rB-mB-) gal*, *dcm* (DE3) | Laboratory stock | | |
| *S. enteritidis* CVCC3377 | | Indicator bacteria strain for determination of MccJ25 antimicrobial activity | Laboratory stock | | |
| *B. subtilis* WB800N | *B. subtilis* 168 derivate, deficient in  *NprE*, *AprE*, *Epr*, *Bpr*, *Mpr*, *NprB*, *Vpr*, *WprA* | | | Laboratory stock |  |
| GWZ08 | WB800N carrying pHT43-MccJ25 | | | Zhang *et al*. (2023) |  |
| GWZ13 | WB800N, Δ*amyE* | | | Zhang *et al*. (2023) |  |
| GWZ16 | WB800N, Δ*amyE*::*mcjABCD* | | | Zhang *et al*. (2023) |  |
| GWZ38 | WB800N, Δ*amyE*::*mcjABCD*, Δ*sigF*::*mcjABCD* | | | Zhang *et al*. (2023) |  |
| GWZ41 | WB800N, Δ*pepA* | | | This study |  |
| GWZ42 | WB800N, Δ*pepF* | | | This study |  |
| GWZ43 | WB800N, Δ*pepT* | | | This study |  |
| GWZ44 | WB800N, Δ*pepA*, Δ*pepF* | | | This study |  |
| GWZ45 | WB800N, Δ*pepA*, Δ*pepT* | | | This study |  |
| GWZ46 | WB800N, Δ*pepF*, Δ*pepT* | | | This study |  |
| GWZ47 | WB800N, Δ*pepA*, Δ*pepF*, Δ*pepT* | | | This study |  |
| GWZ48 | GWZ41 carrying pHT43- MccJ25 | | | This study |  |
| GWZ49 | GWZ42 carrying pHT43- MccJ25 | | | This study |  |
| GWZ50 | GWZ43 carrying pHT43- MccJ25 | | | This study |  |
| GWZ51 | GWZ44 carrying pHT43- MccJ25 | | | This study |  |
| GWZ52 | GWZ45 carrying pHT43- MccJ25 | | | This study |  |
| GWZ53 | GWZ46 carrying pHT43- MccJ25 | | | This study |  |
| GWZ54 | GWZ47 carrying pHT43- MccJ25 | | | This study |  |
| GWZ55 | GWZ16, Δ*sigF* | | | This study |  |
| GWZ56 | GWZ38, Δ*pepA* | | | This study |  |
| GWZ57 | GWZ38, Δ*pepF* | | | This study |  |
| GWZ58 | GWZ38, Δ*pepT* | | | This study |  |
| GWZ59 | GWZ57 carrying pHT43-PepF | | | This study |  |
| BL21O | *E. coli* BL21(DE3) carrying pET28a(+) | | | This study |  |
| BL21A | *E. coli* BL21(DE3) carrying pET28a-PepA | | | This study |  |
| BL21F | *E. coli* BL21(DE3) carrying pET28a-PepF | | | This study |  |
| BL21T | *E. coli* BL21(DE3) carrying pET28a-PepT | | | This study |  |

**Table S2.** Primers and DR sequences used in this study.

| **Primers/ DR** | **Sequence (5’ to 3’)** | **Description** |
| --- | --- | --- |
| pepA-up-F | CAGCACAATCAGCACCTTAGAC | Upstream homology arm of *pepA* gene |
| pepA-up-R | GAAGCCGTGTATGTTGTAACAG |  |
| DR-pepA-F | CTGTTACAACATACACGGCTTC | DR*–spe–upp*–DR (*pepA*) cassette amplification |
| DR-pepA-R | CTTGCTTCCTATGAGGTGCAG |  |
| pepA-down-F | CTGCACCTCATAGGAAGCAAGCATTGCACCGGTTGTTTCTG | Downstream homology arm of *pepA* gene |
| pepA-down-R | CAAATTCGGCTAGAACGCTTG |  |
| DR-pepA | CTGTTACAACATACACGGCTTCAATTTGTTTGTCAGGCTCATTTGATTTATGCTTATAATCCTGCACCTCATAGGAAGCAAG | DR sequence of *pepA* gene |
| pepF-up-F | CACTCAGGTCCGAATGCAGC | Upstream homology arm of *pepF* gene |
| pepF-up-R | GGATTCGTGCCGTAAGCTCC |  |
| DR-pepF-F | GGAGCTTACGGCACGAATCC | DR*–spe–upp*–DR (*pepF*) cassette amplification |
| DR-pepF-R | CAAACTCGTGCACGAGC |  |
| pepF-down-F | GCTCGTGCACGAGTTTGCGGGAAACCTGTATACACAGGC | Downstream homology arm of *pepF* gene |
| pepF-down-R | GCTTCCTCGTATGTCACCTTC |  |
| DR-pepF | GGAGCTTACGGCACGAATCCGTATATTTTGATGAACTGGCATAATAACGTCAATAATCTCTTTACGCTCGTGCACGAGTTTG | DR sequence of *pepF* gene |
| pepT-up-F | CCACCTTTGACACGGAAGAATAC | Upstream homology arm of *pepT* gene |
| pepT-up-R | GAAGTTCTTCAACGGCACGC |  |
| DR-pepT-F | GCGTGCCGTTGAAGAACTTC | DR*–spe–upp*–DR (*pepT*) cassette amplification |
| DR-pepT-R | CTCATGTTGTAGTATTGGTCGTTC |  |
| pepT-down-F | GAACGACCAATACTACAACATGAGGCTTTCTCGCTCACGTCGAC | Downstream homology arm of *pepT* gene |
| pepT-down-R | CTCCTTGAATCGACAGCAGG |  |
| DR-pepT | GCGTGCCGTTGAAGAACTTCAAAACGAATACGGGCAAGACCGGATTCTGCTGGATATGAACGACCAATACTACAACATGAG | DR sequence of *pepT* gene |
| pepA-F | CGCAAGTGTAGCCAGTGTTC | To confirm the deletion of the *pepA* gene |
| pepA-R | TCTTCTGCGGGTATGAAACG |  |
| pepF-F | GGCTTGAATCAAAGAAGACGAGG | To confirm the deletion of the *pepF* gene |
| pepF-R | CGTTTGTCTGAAGACCGTTCC |  |
| pepT-F | CCATTCGAAACGGTATTGTGC | To confirm the deletion of the *pepT* gene |
| pepT-R | GACCATGTTGTCAACTGAGATG |  |


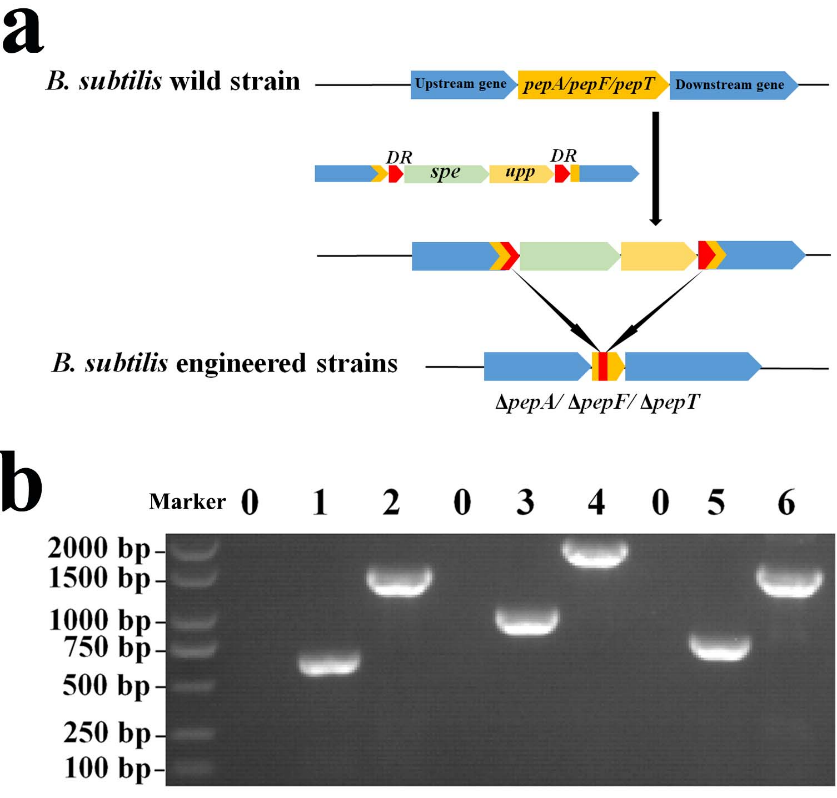


**Fig. S1** Construction of the *B. subtilis* peptidase gene deletion strains. **(a)** Schematic representation of the gene integration based on double-crossover homologous recombination system. **(b)** Genome manipulation of GWZ56, GWZ57 and GWZ58 strains were confirmed by PCR and visualized on an 1% agarose gel. PCR to confirm the deletion of peptidase genes, *pepA* loci use primers pepA-F and pepA-R, *pepF* loci use primers pepF-F and pepF-R, *pepT* loci use primers pepT-F and pepT-R. Lane 0, controls; lane 1, GWZ56 strain in *pepA* loci; lane 2, *B. subtilis* WB800N in *pepA* loci; lane 3, GWZ57 strain in *pepF* loci; lane 4, *B. subtilis* WB800N in *pepF* loci; lane 5, GWZ58 strain in *pepT* loci; lane 6, *B. subtilis* WB800N in *pepT* loci.


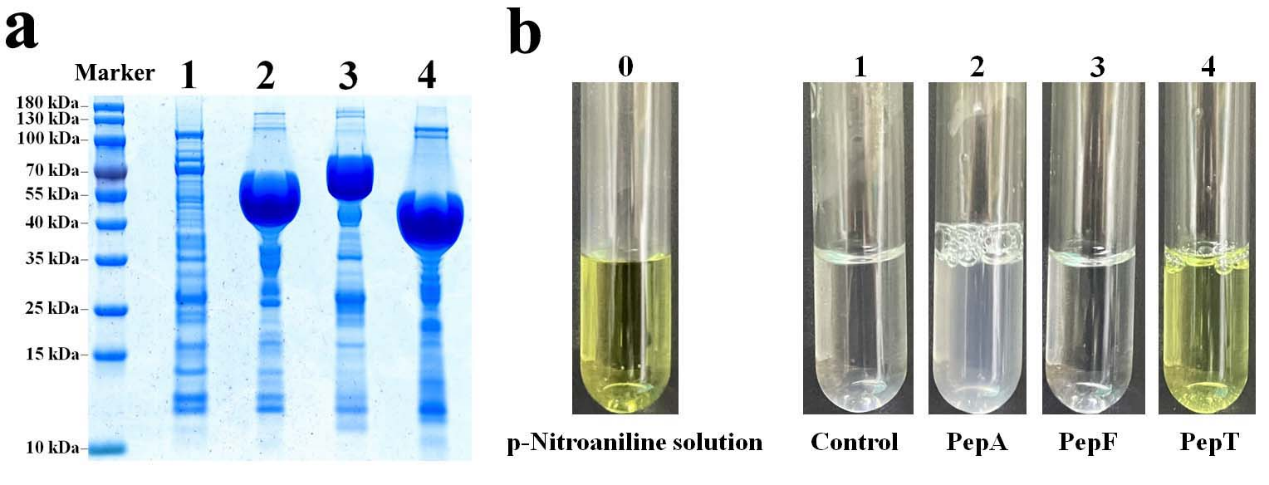


**Fig. S2** Expression and characterization of peptidases. **(a)** SDS-PAGE analysis of peptidases PepA, PepF and PepT successfully expressed in *E. coli* BL21(DE3). Lane 1, control (expression product of BL21O (BL21, pET28a) strain); lane 2, PepA (expression product of BL21A (BL21, pET28a-PepA) strain); lane 3, PepF (expression product of BL21F (BL21, pET28a-PepF) strain); lane 4, PepA (expression product of BL21T (BL21, pET28a-PepT) strain). **(b)** Determination of the peptidase properties of PepA, PepF and PepT using L-leucine-p-nitroaniline. Tube 0, p-nitroaniline standard solution (1 mM); Tube 1, control (expression product of BL21O strain + 10 mM L-leucine-p-nitroaniline); Tube 2, (PepA + 10 mM L-leucine-p-nitroaniline); Tube 3, (PepF + 10 mM L-leucine-p-nitroaniline); Tube 4, (PepT + 10 mM L-leucine-p-nitroaniline).


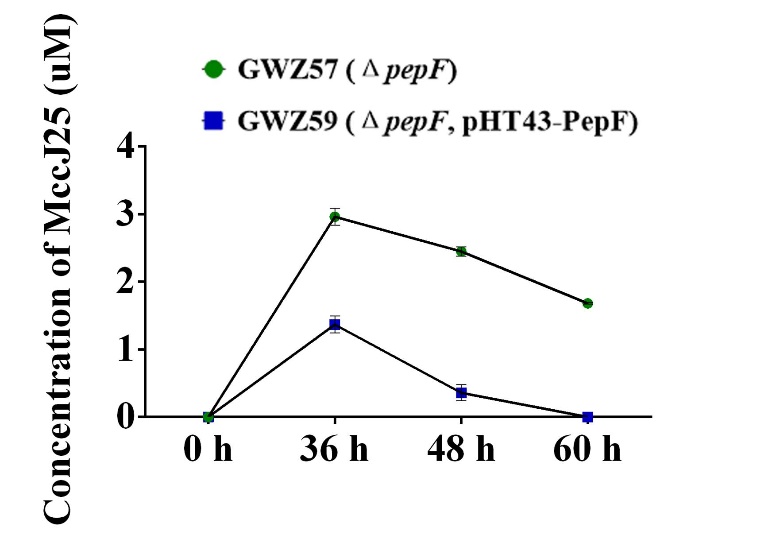


**Fig. S3** The effect of overexpressing *pepF* on MccJ25 expression in *B. subtilis*. The MccJ25 expression levels of GWZ57 (GWZ38, Δ*pepF*) and GWZ59 (GWZ38, *ΔpepF*, pHT43-PepF) strains in the shake-flask culture within 60 h.


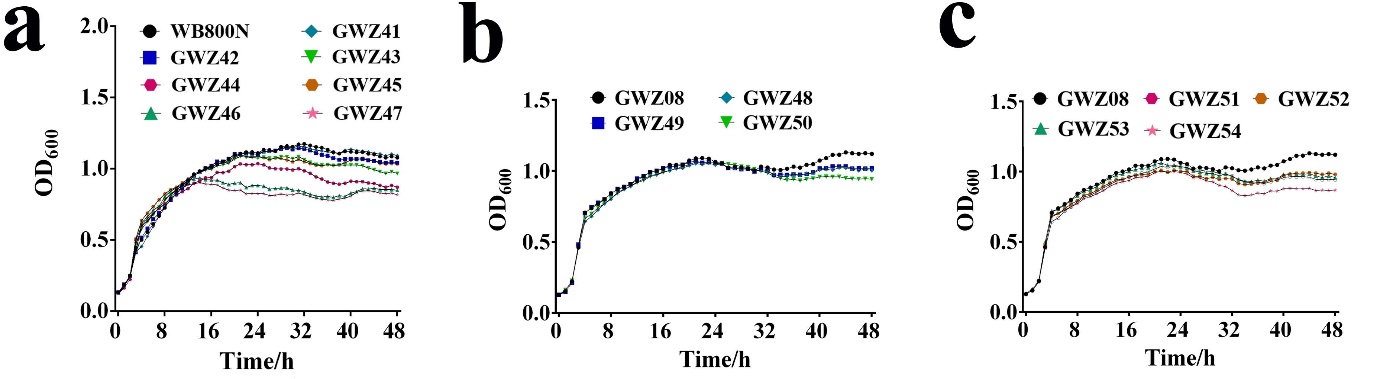


**Fig. S4** Determination of cell growth performance of *B. subtilis* engineered strains. **(a)** Effect of deletion of one or more peptidase genes (*pepA*, *pepF* and *pepT*) on growth performance of *B. subtilis* WB800N strain. GWZ41 (WB800N, Δ*pepA*), GWZ42 (WB800N, Δ*pepF*), GWZ43 (WB800N, Δ*pepT*), GWZ44 (WB800N, Δ*pepA*, Δ*pepF*), GWZ45 (WB800N, Δ*pepA*, Δ*pepT*), GWZ46 (WB800N, Δ*pepF*, Δ*pepT*) and GWZ47 (WB800N, Δ*pepA*, Δ*pepF*, Δ*pepT*). **(b)** Cell growth performance of the *B. subtilis* engineered strains GWZ08 (WB800N, pHT43-MccJ25), GWZ48 (GWZ41, pHT43-MccJ25), GWZ49 (GWZ42, pHT43-MccJ25), GWZ50 (GWZ43, pHT43-MccJ25). **(c)** Cell growth performance of the *B. subtilis* engineered strains GWZ08, GWZ51 (GWZ44, pHT43-MccJ25), GWZ52 (GWZ45, pHT43-MccJ25), GWZ53 (GWZ46, pHT43-MccJ25), GWZ54 (GWZ47, pHT43-MccJ25). All data were the average of five independent experiments with standard deviations.
